# A Case of Spondyloarthritis in Patient Affected by Unicentric Castleman's Disease Effectively Managed with Surgery Resection and Tocilizumab Treatment

**DOI:** 10.1155/2018/5067239

**Published:** 2018-01-23

**Authors:** M. Filippini, S. Cartella, O. Bonzanini, E. Morello, A. Tincani

**Affiliations:** ^1^Unità Operativa di Reumatologia, Spedali Civili di Brescia, Brescia, Italy; ^2^Unità Operativa di Medicina Interna, Azienda Ospedaliera di Desenzano del Garda, Desenzano del Garda, Italy; ^3^Unità Operativa di Ematologia, Spedali Civili di Brescia, Brescia, Italy; ^4^Università degli Studi di Brescia, Brescia, Italy

## Abstract

A 38-year-old woman was referred to our hospital for rheumatologic manifestations (migrant arthritis and tenosynovitis), without psoriasis or family history of psoriasis, gastroenteric manifestations, or recent genitourinary infections. The instrumental and laboratory tests have suggested a diagnosis of undifferentiated seronegative HLA-B27-positive spondyloarthritis with predominantly peripheral involvement. The symptoms were very severe and resistant to anti-inflammatory drugs and steroids. She had a history of hyaline-vascular unicentric Castleman's disease (HBV, HIV, and HHV-8 negative) treated with surgery resection. After a first pharmacological attempt with sulfasalazine (suspended for urticarial rash), we managed the patient with monotherapy tocilizumab 8 mg/kg, with full response of rheumatologic manifestations. The efficacy of tocilizumab was confirmed even after a follow-up of three years. Our experience seems to describe a new late-onset autoimmune disease (only 21 cases described in literature) potentially related to Castleman's disease. The patient experienced marked improvement from IL-6-based therapy (tocilizumab).

## 1. Introduction

Castleman's disease (CD) is a rare polyclonal B lymphocyte proliferation often associated with clinical conditions implying chronic antigenic stimuli-like autoimmune or infectious diseases (i.e., HIV or HHV-8 virus) [1]. CD can be unicentric (U-CD), usually restricted to the mediastinum, or multicentric (M-CD): the first is typically identified incidentally or though symptoms from the local mass effect; the latter form is more symptomatic, including fever, night sweats, weight loss, and anorexia. Moreover, M-CD is the form most commonly associated with autoimmune diseases (AD). The diagnosis of AD can precede or occur contemporaneous or after that of CD (Table 1) [2–7]. Lymph node histopathology is required for diagnosis and to exclude other diseases, like IgG4-related disease or malignant mass (Table 2).

## 2. Case Report

We describe the case of a 38-year-old woman, with a recent history of CD, presented with rheumatologic manifestations (migrant arthritis and tenosynovitis), suggesting the diagnosis of spondyloarthritis (SpA) with predominantly peripheral involvement.

Mediastinum CD was diagnosed incidentally 14 years before, and she underwent surgery resection in 2012 due to the volumetric increase and mass effect. The instrumental staging, performed before the surgical treatment, confirmed the unicentric site. The histological features of the surgical specimen revealed small and atrophic germinal centres, with penetrating hyalinized vessels and follicular dendritic cell (CD21+) expansions; the mantle zones were only partially preserved, whereas the interfollicular region was rich in small T lymphocytes, blood vessels, and plasmacytoid dendritic cells (CD123+). The immunohistochemical technique did not reveal an increase in IgG4 antibody expression compared with total immunoglobulins. Therefore, the histological diagnosis was a CD, hyaline-vascular variant.

At that time, the patient did not experience systemic symptoms like fever, weight loss, anorexia, or arthralgia.

Over the following months (2013), the patient experienced recurrent episodes of hand extensor tenosynovitis, right Achilles enthesitis, and migratory arthritis at right knee and ankle joints. Moreover, US examination revealed a dactylitis of 2nd right finger (flexor tenosynovitis with associated subcutaneous edema). The patient had mechanic low back pain (MRI scan excluded sacroiliitis in T2-weighted image); The HAQ (Health Assessment Questionnaire) was 2.125. Blood tests showed an increase in inflammatory markers; conversely, RF (rheumatoid factor), ACPAs (anti-citrullinated peptide antibodies), and ANAs (anti-nuclear antibodies) were absent; serum uric acid was normal. HIV, HCV, and HBV serologic tests were negative; HLA-B27 antigen was present.

The patient's history and the family history were free from psoriasis, gastroenteric manifestations, or recent genitourinary infections. Moreover, the patient was affected by the following comorbidities: essential hypertension, chronic gastritis, congenital facial angioma treated with sclerotherapy, and cervical intraepithelial neoplasia (CIN1).

Therefore, we have made the diagnosis of seronegative HLA-B27-positive spondyloarthritis (SpA) with predominantly peripheral involvement, according to the current classification criteria [8].

The treatment with anti-inflammatory drugs and steroids was only partially and temporarily effective. Moreover, the patient experienced a drug hypersensibility to the sulfasalazine (urticarial rash). So, we have considered targeted therapies.

## 3. Result

Under our own direct responsibility and after informing the patient and obtaining her consent, in agreement with the Italian Medicines Agency (AIFA), the patient was treated with monotherapy tocilizumab (TCZ) 8 mg/kg as a single intravenous drip infusion administered at 2-week intervals for 4 months (Japanese RCP) and subsequently once a month for 4 months (compassionate use). After a few weeks of treatment, the frequency of articular and periarticular flares was reduced, the inflammatory markers normalized, and the quality of life significantly improved (Table 1). Currently, the patient has been taking the drug for over three years, remaining in clinical, instrumental, and laboratory remission. Both CD and SpA are silent; furthermore, the patient had no adverse events to the drug.

## 4. Discussion

This is the first case reported in literature about a patient affected by SpA, preceded by CD. Our clinical case has suggested some questions: Is there a correlation between the two diseases (CD and SpA)? Was the biologic treatment the best choice for this patient?

As to the first, some data available in literature seem to discourage this hypothesis. CD is often associated with autoimmune diseases; a recent review showed that connective tissue diseases (systemic lupus erythematosus, scleroderma, Sjogren's syndrome, polymyositis, and mixed connective diseases) and, less frequently, chronic arthritis can precede or occur contemporaneously with CD (Table 1 [2–7]). In our case, the patient experienced inflammatory joint involvement about 1 year after the surgical excision and certainly many years after the onset of the haematological disease. The data presented in literature tell us that if the autoimmune disease does not precede the diagnosis of CD usually, the two pathologies are resolved after surgical excision [9]. The fact that this did not happen raises the suspicion that there is not a cause-effect relationship between the two diseases.

However, these considerations are the results of only 21 cases of autoimmune disease associated with CD reported in the literature.

An interesting point of view is the full response to the biologic treatment (ex adjuvantibus). IL-6 elevation in CD was higher than that in other disorders and is considered the most important mediator in the disease, moreover in multicentric form. Above all, in idiophatic multicentric form (iMCD) HBV, HIV, and HHV-8 negative, some studies linked local production of IL-6 to the systemic manifestations of CD. The cells within the lymph node responsible for production of IL-6 have remained elusive; certainly, this cytokine mediates the increase of platelets and fibrinogen and stimulates B cells and TH17 immune profile [10]. In mice, transgenic expression of IL-6 induces a syndrome similar to CD, with lymphadenopathy, splenomegaly, anemia, plasma cell infiltration of lymphoid tissues, and hypergammaglobulinemia [11]. So, TCZ is effective in CD [12].

The blockade of IL-6 is ineffective in axial involvement of SpA and in patients with peripheral arthritis demonstrated contradictory results [13]. Despite two controlled clinical trials have failed to show the efficacy of IL-6 inhibition in ankylosing spondylitis, some experimental studies and case reports [14] suggest a potential therapeutic role for TCZ in patients with SpA with peripheral involvement.

Another peculiarity of this case is the age of onset of CD (24 years). Despite HBV-, HIV-, and HHV-8-negative CD affects people of all ages, it appears to be rare in children and younger. The mean age of patients with M-CD is 50–65 years; males account for 50–65% of all cases [15].

## 5. Conclusion

In conclusion, our experience seems to describe a new late-onset autoimmune disease potentially related to a rare haematological disease (CD). The patient experienced marked benefit from IL-6-based therapy (TCZ).

## Figures and Tables

**Table 1:** Cases of AD associated with CD [2–7].

AD associated with CD (*n* = 21)	U-CD (*n* = 9)	M-CD (*n* = 12)	AD preceded CD (*n* = 6)	CD preceded or occurred contemporaneous with AD (*n* = 15)
Myasthenia gravis	6	1	0	7
SLE	0	4	0	4
Systemic sclerosis	1	0	0	1
Sjogren's syndrome	1	2	2	1
Polymyositis	0	1	1	0
Undifferentiated CTD	0	1	1	0
Mixed CTD	0	2	0	2
Rheumatoid arthritis	1	1	2	0

**Table 2:** Principal laboratory and clinical parameters of the patient before and after TCZ treatment.

	Baseline	4th month	12th month	24th month	36th month
PCR (mg/l)	8.5	1	1	1	1
VES (mm/1h)	48	12	11	15	13
DAS28	4.25	1.7	1.5	1.8	1.2
SDAI	27.85	4.1	3.3	3.2	2.9
CDAI	27	4	3.2	3.1	2.8
HAQ	2.125	1	0.75	0.75	0.75
